# Characterization of novel elongated Parvulin isoforms that are ubiquitously expressed in human tissues and originate from alternative transcription initiation

**DOI:** 10.1186/1471-2199-7-9

**Published:** 2006-03-07

**Authors:** Jonathan Wolf Mueller, Daniel Kessler, Daniel Neumann, Tina Stratmann, Panagiotis Papatheodorou, Cristina Hartmann-Fatu, Peter Bayer

**Affiliations:** 1 Department Structural and Medicinal Biochemistry, Center for Medical Biotechnology – ZMB, University of Duisburg and Essen, 45117 Essen, Germany; 2 Institute for Microbiology, University Hohenheim, Garbenstraße 30 (Bio-Zentrum), 70599 Stuttgart, Germany

## Abstract

**Background:**

The peptidyl prolyl cis/trans isomerase (PPIase) Parvulin (Par14/PIN4) is highly conserved in all metazoans and is assumed to play a role in cell cycle progression and chromatin remodeling. It is predominantly localized to the nucleus and binds to chromosomal DNA as well as bent oligonucleotides *in vitro*.

**Results:**

In this study we confirm by RT-PCR the existence of a longer Parvulin isoform expressed in all tissues examined so far. This isoform contains a 5' extension including a 75 bp extended open reading frame with two coupled SNPs leading to amino acid substitutions Q16R and R18S. About 1% of all Parvulin mRNAs include the novel extension as quantified by real-time PCR. The human Parvulin promoter is TATA-less and situated in a CpG island typical for house keeping genes. Thus, different Parvulin mRNAs seem to arise by alternative transcription initiation. N-terminally extended Parvulin is protected from rapid proteinaseK degradation. In HeLa and HepG2 cell lysates two protein species of about 17 and 28 KDa are detected by an antibody against an epitope within the N-terminal extension. These two bands are also recognized by an antibody towards the PPIase domain of Parvulin. The longer Parvulin protein is encoded by the human genome but absent from rodent, bovine and non-mammalian genomes.

**Conclusion:**

Due to its molecular weight of 16.6 KDa we denote the novel Parvulin isoform as Par17 following the E. coli Par10 and human Par14 nomenclature. The N-terminal elongation of Par17-QR and Par17-RS suggests these isoforms to perform divergent functions within the eukaryotic cell than the well characterized Par14.

## Background

Members of the parvulin family of peptidyl prolyl cis/trans isomerases (EC 5.2.1.8) are involved in mitotic regulatory mechanisms and cell proliferation [1-4]. The human genome only encodes two parvulin proteins – Pin1 and Parvulin. Pin1 is a well studied mitotic regulator involved in cell cycle and transcriptional regulation [1,4,5]. Overexpression of Pin1 was found in several tumor types [6,7]; inhibition of this enzyme leads to apoptosis in a Ras-transformed cell line [8]. The second member of the parvulin PPIase family, the 14 KDa protein Parvulin14 [9,10] (Par14 or PIN4), is assumed to be involved in cell cycle progression or chromatin remodeling [11,12]. Pin1 and Par14 sequences are found in all multi-cellular organisms from *N. crassa *and *C. elegans *to man, whereas yeasts contain only one parvulin homolog called Ess1. The lethal phenotype of Ess1 deletion can only be rescued by human Pin1 but not by hPar14 [13] suggesting divergent cellular functions for the two human parvulins.

Although the C-terminal PPIase domains of human Pin1 and Par14 proteins are similar both in sequence and in three-dimensional structure, their N-terminal domains differ significantly. Whereas hPin1 carries an N-terminal WW motif [2,5,14], the basic domain of hPar14 (comprising the first 35 amino acids) is unfolded in solution [15]. Par14 is localized in cytosol and nucleus with deletion studies pointing to a nuclear import signal located within the basic N-terminal part [12]. Within the nucleus, Par14 was reported to bind to pre-ribosomal ribonucleoprotein particles [16], and sequence specifically to bent double stranded DNA [12]. Such bent A/T rich segments of DNA are supposed to dictate nucleosome positioning and play a role in transcription initiation. The N-terminal part of Par14 is necessary for high affinity DNA binding. Phosphorylation within this region at Ser19 regulates nuclear localization and DNA binding [11].

Within the N-terminal basic domain Par14 shows 45% homology to the region surrounding the chromatin unfolding domain of HMGN proteins [12,15], whose residues are involved in contacts to nucleosomal DNA. A structural feature called the HMGB domain which is well known from transcription factors LEF-1 or SRY can be found as part of the catalytic domain within the first alpha-helix of the PPIase domain of Parvulin [12]. This motif includes a hydrophobic patch (Ile51, Met52 and Met55 in Par14) that conforms to a minor groove binding mode. For the transcription factor LEF-1 these residues were shown to be essential for contacting DNA within the minor groove and for inducing bent DNA structures.

Despite all these data, there are several open questions concerning the cellular function of Parvulin. In this study we confirm by RT-PCR the existence of a longer Parvulin isoform that has an extension at the 5' end including a 75 bp extended open reading frame. As *E.coli *Par10 and human Par14 are numbered according to their molecular weight, we refer to the novel Parvulin isoform as Par17.

## Results and discussion

### Identification and quantification of new isoforms of human Parvulin mRNA

**Figure 1:**
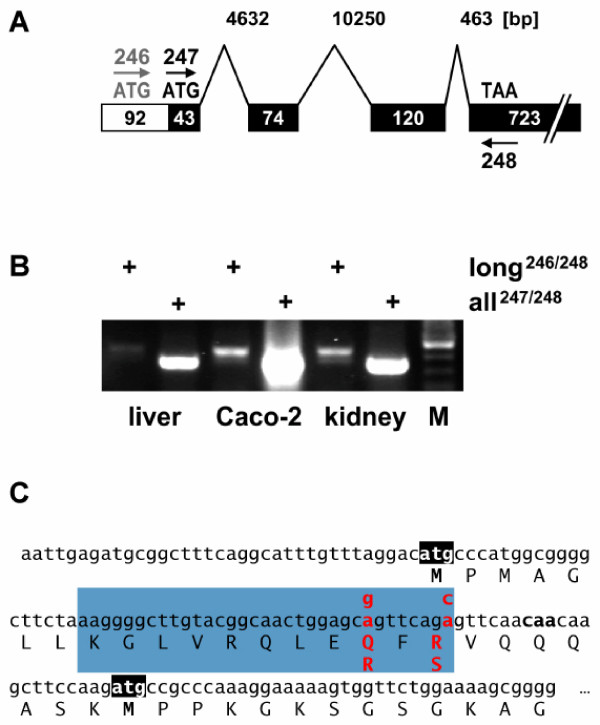
**A – Schematic representation of the human Parvulin gene**. The mRNA is spliced together from four exons between 43 and 723 bp in length. The size of all introns and exons is given in base pairs (bp). The longer Parvulin mRNA contains a 92 bp extension at the 5' side depicted in white. Primers used for RT-PCR are indicated by arrows. Primers 246 and 247 are complementary to the sequences around the two start ATG codons, 248 binds at the sequence around the TAA stop codon. **B – RT-PCR products from different cDNA samples**. RT-PCR products with mRNA from liver, kidney and Caco-2 cells after 30 amplification cycles. Primers 246 and 248 yield a 488 bp PCR product only on those Par14 mRNAs with 5' extension. Primers 247 and 248 give rise to a 385 bp DNA fragment with all Par14 mRNAs. All longer RT-PCR products were eluted from the gel and sequenced. **C – First 150 nucleotides of the elongated Parvulin mRNA**. Two start ATG codons are indicated in bold capital letters. The sequence of Parvulin common to both originally described cDNAs by Uchida *et al*. [GenBank:AF143096] [3] and Rulten *et al*. [GenBank:AB009690] [9] begins at the caa codon depicted in bold. The peptide sequence used for antibody production is shaded in grey. Two SNPs are shown leading to amino acid substitutions Q16R and R18S.

Recently, the Parvulin RefSeq [17] entry for human Parvulin [GenBank:NM_006223.2] was updated by incorporating a 5' extension of about 90 nucleotides relative to the original database entries [9,10]. This sequence was only encoded by one expressed sequence tag (EST) [GenBank:AU098526]. To prove this extension to occur within cells, we performed RT-PCR on human mRNAs from different tissues. As the 5' extension contains an additional start codon 75bases upstream of the original start codon, primers were designed to confirm the occurrence of both the short Par14 and the extended open reading frames by RT-PCR. A schematic representation of the Parvulin mRNA together with the positioning of primers is given in Figure 1A. The PCR products corresponding to the 5' extension and total Parvulin mRNA were detected in all tissues tested so far. The PCR signal for the 5' extension is much weaker than the total Parvulin PCR product. RT-PCR bands from liver, kidney and Caco-2 cDNAs are shown exemplarily in Figure 1B.

All 488 bp DNA fragments corresponding to the elongated Parvulin mRNA were eluted from gels and sequenced. Thereby, two nonsynonymous single nucleotide polymorphisms (SNPs) leading to two amino acid substitutions Q16R and R18S were detected within the 5' extension that are also referenced in the NCBI SNP database as rs6525589 and rs7058353, respectively. Out of 14 different cDNAs sequenced, only two did contain these mutations, the liver and kidney cDNA samples used in this study. We only have observed the coupled occurrence of these two SNPs. In addition, these two cDNAs had the silent GCC to GCT mutation at Ala93 (rs16991466) within the known part of the Par14 mRNA. The first 176 nucleotides of the elongated Parvulin mRNA together with the detected coupled SNPs are depicted in Figure 1C.

**Figure 2:**
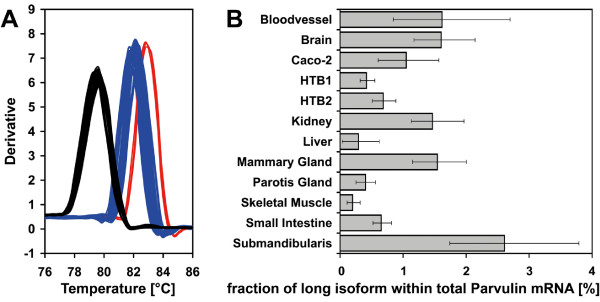
**Real-time PCR quantification of human parvulin mRNA isoforms**. **A**. Dissociation curves for total parvulin mRNA (total) and long isoforms (indicated with QR and RS). **B**. Fraction of long parvulin mRNA isoform within amount of total Parvulin mRNA in cDNAs from the tissues indicated.

To quantify relative amounts of different Parvulin mRNA isoforms, real-time PCR primers that detect the novel 5' extension and total Parvulin mRNA (indicated in Figure 1A) were designed and confirmed to yield uniform PCR products by agarose gel electrophoresis. mRNAs from different sources were extensively treated with DNase to degrade any traces of genomic DNA and reverse transcribed. The above mentioned primers were then used to perform real-time PCR with SYBR Green detection on the resulting cDNAs to analyze the extended and total Parvulin messages. The melting curves of the generated PCR products are shown in Figure 2A. Therein the maxima represent the melting points of the corresponding PCR products with 79.5°C for the fragment corresponding to total Parvulin mRNAs. The melting curves for the PCR products originating from the Parvulin 5' extension can be grouped into two ensembles with about 82°C and 83°C melting temperatures. The former corresponds to the above mentioned QR variant; the elevated melting point of the latter can be explained by two AT base pairs changed to GC bases in the RS variant. Thus, the primers used in this study should enable fast real-time SNP detection of RS and QR genotypes in other human cDNAs or patient samples.

Relative amounts of long Parvulin mRNA and total Parvulin mRNA were then calculated based on the real-time PCR data. The fraction of the long isoform within total Parvulin mRNA is given in Figure 2B ranging from 0.2% in the skeletal muscle cDNA sample used for this study to 2.6% in a cDNA from the submandibularis gland.

### An elongated Parvulin isoform is expressed in human cell lines

**Figure 3:**
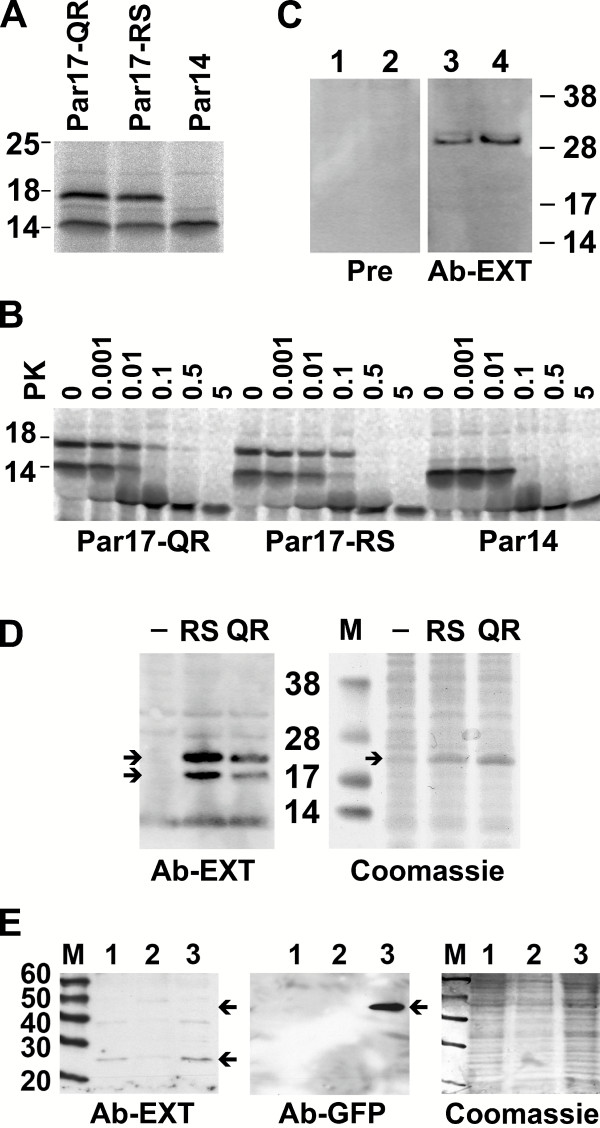
**A – *In vitro *translation of Parvulin isoforms**. Open reading frames for Par17-QR, Par17-RS and Par14 were TOPO-cloned into the vector pCR-4 and 35S-methionine labeled in an *in vitro *transcription/translation reaction. Reaction products were separated by 17.5% SDS-PAGE following autoradiography. **B – Proteinase K digestion of Parvulin isoforms**. Equal amounts of 35S-methionine labeled Par17-QR, Par17-RS and Par14 were incubated with increasing concentrations of proteinaseK (PK) for 30 min at 10°C. Enzyme concentrations are given in μg/ml. Reactions were stopped by an excess of PMSF, separated by SDS-PAGE and subjected to autoradiography. **C – Western blots of cell lysates**. Western blot of HepG2 (lane 1 and 3) and HeLa (lane 2 and 4) cell lysates. Proteins were separated by SDS-PAGE with MES as running buffer and SeeBlue2 as protein standard, transferred to nitrocellulose membranes. Blots were incubated with pre-immune (lane 1 and 2) or anti-Par17 serum (lane 3 and 4; both at 500-fold dilution). **D – The newly raised antibody Ab-EXT recognizes Par17-RS and -QR**. Coding sequences for Par17-RS and -QR were subcloned in the pET-28 vector with N-terminal His6 tag and expressed in *E. coli*. Lysates were subjected to reducing SDS-PAGE in MES buffer and SeeBlue2 as protein standard, transferred to nitrocellulose membranes and incubated with the anti-Par17 antibody. -, before IPTG induction; RS, Par17-RS lysates; QR, Par17-QR lysates. Coomassie stained gel of lysates to show equal loading. Par17 fusions with His6 tag and thrombin cleavage sites show apparent molecular weights of about 22 KDa in SDS-PAGE. The induction band in E. coli lysates and the corresponding band recognized by Ab-EXT are labeled with arrows. The lower migrating band may be caused by proteolytic degradation. **E – Ab-EXT does not recognize GFP-Par14**. Par14 coding sequence was expressed as GFP fusion in HeLa cells (lane 3). Lane 1 and 2 are HeLa cell lysates not transfected with this construct. Lysates were subjected to reducing SDS-PAGE in Tris-glycine buffer with MagicMark as protein standard, transferred to nitrocellulose membranes and incubated with anti-Par17 and anti-GFP antibodies. Coomassie stained gel of lysates to show equal loading.

As not every ATG codon is necessarily used for translation initiation, we attempted to translate the different coding sequences of Parvulin *in vitro*. Therefore, the open reading frames for the QR and RS Parvulin variants as well as Par14 were TOPO-cloned into the vector pCR-4 and 35S-methionine labeled in a T7 polymerase/reticulocyte *in vitro *transcription/translation system Figure 3A. A 14KDa translation product was produced both from the short and elongated Parvulin templates suggesting that the context of the second ATG is suited for translation initiation despite the lack of a classical Kozak sequence [18]. In addition, QR and RS Parvulin templates yielded a translation product of about 17KDa in agreement with a theoretical molecular weight of 16,6 KDa. Therefore, we refer to the novel Parvulin isoform from now on as Par17 according to the denotation of other parvulins [10,19]. Although Parvulin is also referred to as PIN4 in databases we prefer the name Par14/Par17 to avoid confusion with a putative auxin efflux carrier protein from *Arabidopsis *that is incidentally named PIN4, too.

The longer Parvulin mRNA encodes a protein N-terminally extended by 25 amino acids. tBLAST searches with this N-terminal protein sequence as query neither yield significant similarities in the nr database of GenBank nor at other loci within the human genome. The extension is predicted to adopt an additional amphipatic alpha-helix in the protein [20]. Rulten *et al. *and Sekerina *et al. *reported fast degradation of the basic and flexible domain of Par14 by Chymotrypsin and an *E. coli *protease, respectively [9,15]. We were interested, whether the amphipatic helix might be able to stabilize the degradable protein part by binding to the catalytic domain or by masking it. Thus, the structural integrity of Par17 was tested by limited proteinaseK digestion. At minor enzyme concentrations (0.01 μg/ml) the 35S-methionine labeled short Parvulin is degraded leaving over the catalytic domain only (Figure 3B). For degradation of the long protein isoforms at least five-fold higher protease concentrations are needed. This indicates that the N-terminal elongation seems to protect the protease sensitive basic region from being digested rapidly.

**Figure 4:**
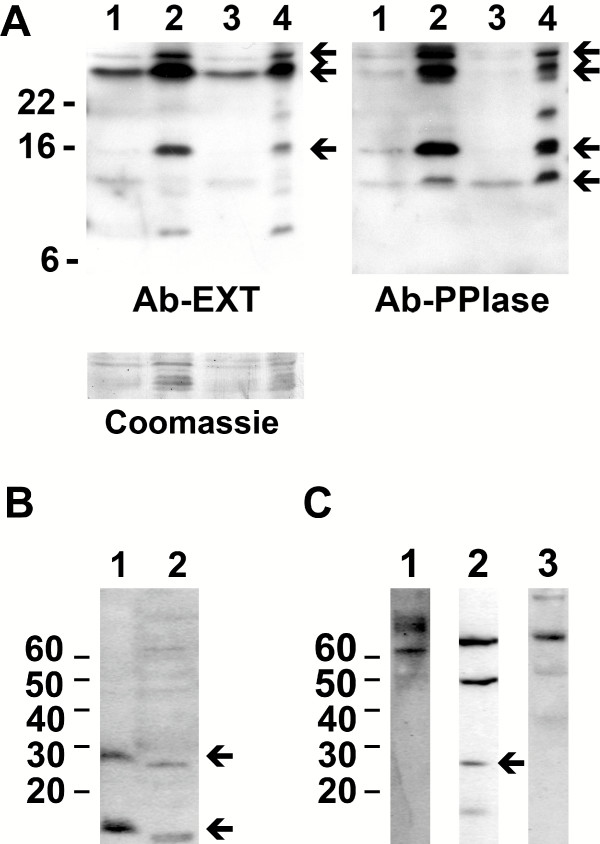
**Detection of Par17 in cell lysates**. **A**. Western blot of HeLa and HepG2 cell lysates. 10 and 50 μg HeLa (lane 1 and 2) and 10 and 50 μg HepG2 (lane 3 and 4) cell extracts were separated by SDS-PAGE with Tris-glycine as running buffer and transferred to nitrocellulose membranes. SeeBlue2 was used as protein standard. Blots were incubated with Ab-PPIase (polyclonal antibody against Parvulin's PPIase domain as described [11,12], stripped by 2% SDS at 65°C and re-probed with affinity purified Ab-EXT (against the N-terminal extension). Coomassie stained membrane is shown as loading control. **B**. Fresh protein samples (lane 1) as well as samples subjected to repeated freeze-thaw cycles (lane 2) were analyzed by Western blots using anti-Par17 antiserum. C; HeLa cell extracts were separated by SDS-PAGE with Tris-glycine as running buffer, transferred to nitrocellulose and incubated with antibodies against SUMO-1 (lane1), SUMO-2/3 (lane2) and Ubiquitin (lane3). Magic Mark was used as protein standard.

Next, we wanted to prove the expression of the elongated Parvulin isoform in human cells. Therefore, a polyclonal antibody was raised against an epitope within the N-terminal extension of human Par14 that was shaded in gray in Figure 1C. This antibody recognized both the Q16/R18 and R16/S18 isoforms of the elongated Parvulin protein overexpressed in *E. coli *with slightly higher affinity towards the RS form (figure 3D) but does not recognize GFP fused Par14 expressed in HeLa cells that lacks the N-terminal extension (figure 3E). Thus, the expression of the elongated protein can be detected irrespective of the particular genotype. In most fresh protein lysates from HeLa and HepG2 cells, only the 28 KDa species was detected (Figure 3C). In addition, we detected a 17 KDa species in other HeLa and HepG2 lysates. After repeated freeze-thaw cycles, this second protein species at 17 KDa gained intensity with the 28 KDa band appearing much weaker (Figure 4B). Despite the presence of protease inhibitors, this observation could be attributed to the already reported high protease sensitivity of Parvulin [9,15].

An antibody against the PPIase domain was used to detect Parvulin isoforms in HepG2 and HeLa cell lysates (figure 4A as described in previous studies [11,12]. This antibody recognized Par14 at about 14 KDa, one protein species with an apparent molecular weight of nearly 17 KDa and two bands larger than 22 KDa indicating that all these bands do contain Parvulin's PPIase domain. This blot was stripped and re-probed with the antibody towards the N-terminal extension that was affinity purified on a CNBr-coupled Par17-QR/RS sepharose column to enhance specificity. Again, a 17 KDa and two protein species above 22 KDa were detected. This antibody did not recognize the Par14 band. This shows that Par17 is present within human cells both in modified and unmodified forms.

The 28 KDa protein species described above could be explained with an SDS stable protein dimer. Such dimers are described for hydrophobic, transmembrane proteins. As extended hydrophobic regions could not be detected in the primary structure of Par17 we excluded the assumption of a Parvulin dimer. Alternatively, the difference of about 10 KDa between in vitro translated Par17 and the 28KDa immunoreactive protein species in cell lysates suggests post-translational modification with a proteinous tag. To test this, Western blots were re-probed with antibodies against SUMO1, SUMO2/3 and Ubiquitin. Neither SUMO1 nor Ubiquitin conjugated species could be detected in the range between 20 and 40 KDa, only one band of 28KDa recognized by the anti-SUMO2/3 antibody overlaid with the Parvulin band suggesting a conjugate between Par17 and SUMO2 or SUMO3 (Figure 4C). As the fast degradation of the 28KDa protein species prevented co-immunoprecipitation studies a final proof for the presence of a Par17-SUMO2/3 conjugate is still missing.

The above mentioned polyclonal antibody against the isolated PPIase domain of Par14 was also used in previously performed Western blot studies of human cell lysates (Surmacz et al. and Reimer et al.). In both studies, there was a protein species detected at 28 KDa in addition to the Par14 band. In addition, Reimer *et al*. applied HEK293 cell lysates to DNA-cellulose; bound proteins were eluted by increasing salt concentrations. Parallel to the detected Par14 band, another protein species was clearly visible at about 28 KDa which we now have identified as modified Par17. Therefore, we propose that Par17 may be able to bind genomic DNA in a similar manner to Par14.

### Transcriptional initiation at the human Parvulin promoter

The present study shows that in addition to the original Par14 protein elongated isoforms of Parvulin are expressed in human cells. The question arises whether these isoforms are encoded by alternative open reading frames. A genome wide BLAST search revealed sequences on chromosome 1 and 15 that show a high degree of sequence similarity to the well known Parvulin locus on chromosome Xq13 with the gene name *PIN4*. As they do not encode an N-terminal elongation and are only 87% (chromosome 1) and 97% (chromosome 15) identical to the described Parvulin sequence, these putative pseudogenes are not responsible for the occurrence of the isoforms in human tissues observed in our studies. Therefore we conclude that the different isoforms must be encoded by the *PIN4 *gene on chromosome Xq13.

The sequence preceding the transcription start site displays an extraordinarily high CpG ratio (0.864) and a GC content of 51.6% (calculated for 200 bp upstream of the first ATG codon), but lacks conserved TATA boxes and downstream promoter elements. Only one sequence motif immediately upstream of the first nucleotides of the longest EST shows some similarity to the consensus initiator sequence [21]. TATA-less promoters within CpG islands are typical for housekeeping genes. They lack one strong promoter but instead contain several weak promoter elements [21] resulting in more than one transcription initiation sites.

Using the extended cDNA sequence for human Parvulin as query, the EST database of GenBank was searched by BLAST [22]. For alignment only EST clones sequenced from their 5' end exceeding an overlap of 50 nt with the original cDNA entry of Uchida et al. were taken [10]. Intron containing ESTs were removed manually from this collection, thereby excluding one 5' extended EST [GenBank:BI915994]. From the remaining 70 database entries only the above mentioned AU098526 encoded the long isoform corresponding to 1.5% of all sequences. This ratio between long and total Parvulin mRNA is in good agreement with our real-time data. Thus, both a short and an extended version of Parvulin mRNA occur within human cells. Taken the short length of Parvulin mRNAs of about 1 kB, we assume most if not all of these EST sequences to be full length. Therefore, we aligned all Parvulin ESTs sequenced from the 5' end which should be indicative for the initiation of transcription at the Parvulin locus. All ESTs differed to a certain extent in their 5' start. These different transcript starting points are in agreement with transcriptional initiation from different weak initiator points within a CpG island promoter that give raise to different Parvulin isoforms.

### Parvulin genes in other organisms

**Figure 5:**
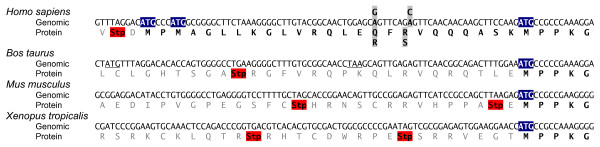
**Alignment of Parvulin genomic DNA sequences from different species**. Genomic sequences from *Homo sapiens*, *Bos taurus*, *Mus musculus *and *Xenopus tropicalis *are aligned with the ATG start codon of Par14 common to all these sequences. The human sequence contains 5' to the Par14 coding sequence two additional in-frame ATG codons (highlighted in blue). The only other 5' ATG codon is present in the *Bos taurus *sequence; it is out of frame and followed by a TAA stop codon (both underlined). All in-frame stop codons are marked in red. Only the human sequence displays an extended open reading frame, both as QR and RS isoform (SNPs highlighted in grey).

The PPIase sequence of Par14 is very well conserved from *N. crassa *and *C. elegans *to man with identities of 54.2% and 82.3% compared to the human sequence. Therefore it was interesting to search for extended Parvulin variants in other organisms as well. Genomic Parvulin sequences were available from 13 different species  which were compared with regard to additional coding regions preceding the Par14 coding sequence. A sequence from *Canis familiaris *more likely resembled the above mentioned Parvulin pseudogene and was not further analyzed. Genomic sequences from chimp and chicken only contained the last two of the four Parvulin exons with otherwise very similar exon length. Despite all efforts, no more upstream sequence information was available for these organisms from internet resources. Even the recently published improved chimp sequence only contains Parvulin exons three and four [23]. With the exception of *C.elegans *and insects, all Parvulin genes were identical in their genomic structure. They all contained one or more additional start codons within an area 200 bp upstream of the Par14 ATG, however nowhere – not even in mammals such as *Mus musculus *or *Bos taurus *– these ATGs resulted in extended open reading frames with similarities to human Par17 (Figure 5). It will be especially interesting to search for Par17 within an improved version of the chimp genome in the near future. In contrast to the ubiquitous occurrence of Par14, the expression of the elongated isoform Par17 seems to be limited to some mammals with proven expression up to now only in *Homo sapiens*.

## Conclusion

Par14 is highly conserved in all metazoans. Human Parvulin mRNA is expressed in all tissues examined so far pointing to ubiquitous expression. The TATA-less Parvulin promoter is situated in a CpG island typical for house keeping genes; such promoters often contain several weak transcription start points. At least in humans this alternative transcription initiation gives raise to multiple mRNA isoforms which could be proven by RT- and real-time PCR. These mRNAs encode the formerly known Par14 as well as N-terminally extended Par17. This longer protein is expressed in HeLa and HepG2 cells and is probably post-translationally modified. Its solution structure and catalytic properties as well as interactions with possible partners can now be addressed.

## Methods

### Bioinformatics

The EST databases of GenBank were searched by BLAST  for EST sequences with 5' extensions relative to the original cDNA entry [GenBank:AB009690] [10]. Therefore, nucleotides 1 to 500 from the RefSeq entry for Parvulin [GenBank:NM_006223] as well as the coding sequence of Par17-RS were used as query. Only 5' clones exceeding an overlap of 50 nt with the original cDNA entry without intronic sequence were aligned within the BioEdit software . Genomic sequences of Parvulin homologs from 13 different species (*Homo sapiens, Mus musculus, Pan troglodytes, Bos taurus, Canis familiaris, Gallus gallus, Tetraodon nigroviridis, Fugu rubripes, Xenopus tropicalis, Caenorhabditis elegans, Drosophila melanogaster, Danio rerio *and *Anopheles gambiae*) were retrieved from the Ensembl genome browser . Exons and neighboring intronic sequences (+ 200 nt) were aligned within the BioEdit software . GC content and CpG ratio were determined for an area of 500 nt surrounding the transcription start site and 500 nt downstream of the stop codon as a reference. The CpG ratio is defined as (observed CG dinucleotides/expected CG dinucleotides). A CpG island according to [24] is defined as a sequence longer than 200 nt with a CpG ratio above 0.6 and a GC content of more than 50%.

### mRNAs and primers for RT-PCR

DNA-free total RNAs for human tissues (kidney, small intestine, brain, blood vessel, mammary gland and skeletal muscle) were purchased from Biocat BioChain Institute (Hayward, CA, USA). cDNAs from parotis and submandibularis glands were kindly provided by Dr. Veronika Homann and liver cDNA by Dr. Frank ter Veld. Total RNA from Caco-2 and HTB cells was isolated using RNAeasy kits (Qiagen, Hilden) according to manufacturer's instructions. Preceding cDNA synthesis with Superscript II reverse transcriptase (Invitrogen, Karlsruhe), all samples were incubated with DNAse to remove any trace of genomic DNA. PCR was performed subsequently using Taq Polymerase (NEB, Frankfurt) with the following primers (5' to 3'): 246, atgcccatggcggggcttctaaag; 247, gaaaagcggggaaagggggagcag and 248, cagtctttcatatgattttattttcttccttcgacc, all showing similar melting points. PCR products were analyzed on agarose gels and eluted bands were sequenced.

### Real-time PCR

To quantify relative amounts of Parvulin transcripts real-time PCR primers were designed using the ABI program Primer Express. To detect the longer transcript the following primers were used (5' to 3'): 251, cggctttcaggcatttgtttag and 252, gcggcatcttggaagcttgtt. Total amount of Parvulin transcripts was detected with primers 253, tgggagtgacagtgctgacaa and 254, catgtttttcacatagaatgtgtctgac. Real-time PCR was performed with SYBR green kits (Qiagen, Hilden) on a GeneAmp 5700 sequence detection system (Applied Biosystems, Perkin Elmer). Uniformity of PCR products was verified by gel analysis and melting curve analysis. Relative PCR efficiencies were determined using dilution series of RT-PCR products as standard. All reactions were performed in triplicate. As Ct values are at a logarithmic scale, they were averaged to their geometric mean values; errors are given as standard deviations.

### Cell lysates, antibodies and Western blotting

HepG2 cells (Tumorbank, DKFZ Heidelberg) were cultivated in RPMI 1640 + 10% FBS, 1% Penicillin/Streptomycin at 37°C and 5% CO_2_. HeLa cells (ATCC, USA) were cultivated in DMEM with 25 mM Glucose + 10% FBS, 1% L-Glutamine, 1% MEM at 37°C and 5% CO_2_. Cells were grown to confluency in 100 mm petri-dishes, washed with PBS and harvested into PBS + protease inhibitor mix on ice (Complete without EDTA, Roche Diagnostics, Mannheim). Pelleted cells after 5 min at 400 × g and 4°C from 4 plates were resuspended in 0.5 ml PBS + Complete, lysed by sonication and frozen at -20°C until use.

Equal amounts of protein were separated on 4 to 12% gradient SDS gels (Invitrogen, Karlsruhe) with MES running buffer including 0.5 M 2-mercaptoethanol and transferred to nitrocellulose membrane. Blocking was done in PBS with 1 or 2% BSA with or without 0,05% Tween. An antiserum (#4113) raised against a mixture of KGLVRQLERFS and KGLVRQLEQFR peptides (SeqLab, Göttingen) was used for detection of Par17 expression at 1:500 dilution in PBS + 1% BSA. Par17-QR and -RS reading frames were subcloned into pET-28 with His6 fusion and thrombin cleavage site. Recombinant proteins were expressed in *E. coli*, purified on Ni-NTA columns and coupled to CNBr-activated sepharose (Sigma) according to the manufacturer's protocol. This Par17 column was used for affinity purification of final bleeding of polyclonal antibody #4113 ("Ab-EXT"). For comparison the polyclonal antibody against Parvulin's PPIase domain was used as described [11,12]. For characterization of Par17's modification rabbit anti-SUMO-1 (Santa Cruz, USA, 1:100 dilution), rabbit anti-SUMO-2/3 (Zytomed, Berlin, 1:50) and mouse monoclonal anti-ubiquitin (Covance, Berkeley, USA, 1:50) antibodies were used at the indicated dilutions. HRP conjugated anti-rabbit and anti-mouse antibodies together with ECL kits (Amersham Bioscience, Freiburg) were used for detection with a chemiluminescent camera (Bio-Rad, Munich). Stripping of Western blot membranes was either done with stripping reagent (Pierce) or two incubation steps in 2% SDS at 65°C.

### *In vitro *translation and proteinase K digestion

For *in vitro *translation experiments the coding sequence for Parvulin isoforms was amplified from RT-PCR products using the primers (5' to 3') 300 F (aaaaaagaattcgccaccatgcccatggcggggcttctaaag) and 258 (acacacctcgagattattttcttccttcgaccataataat) for Par17-RS and Par17-QR forms; Par14 was amplified with primers 304 F (aaaaaagaattcgccaccatgccgcccaaaggaaaaagtggt) and 258 (Kozak sequence within forward primers 300 F and 304 F is underlined). PCR products were TA TOPO cloned into pCR-4 (Invitrogen, Karlsruhe). Correct orientation of inserts relative to the T7 promoter was verified by restriction analysis and DNA sequencing. The resulting constructs were used for *in vitro *transcription and translation reactions with ^35^S-methionine incorporation (ICN, Eschwege, Germany) using the coupled TNT reticulocyte lysate system (Promega, Mannheim) according to the manufacturer's instructions. The lysates were separated on 17.5% SDS gels following autoradiography. For limited proteolysis studies, lysates were incubated with varying concentrations of proteinaseK at 10°C for 30 min, reactions were stopped by an excess of PMSF and also analyses by SDS-PAGE and autoradiography.

## Abbreviations

EST, expressed sequence tag; Par17, Parvulin 17; PCR, polymerase chain reaction; RT, reverse transcriptase; SNP, single nucleotide polymorphism; bp, base pars; nt, nucleotide(s); Ab-EXT, polyclonal antibody against Parvulin's N-terminal extension; Ab-PPIase, polyclonal antibody against Parvulin's PPIase domain

## Authors' contributions

JWM conceived and coordinated the study, carried out real-time PCR, analyzed modifications of Parvulin and drafted the manuscript. DK carried out retrieval and analysis of EST and genomic sequences and participated in writing. TS performed RT-PCR and participated together with DN in cloning and Western blotting. PP carried out *in vitro *translations and performed limited proteolysis studies together with JWM. CHF participated in Western blotting and contributed to writing the manuscript. PB participated in design and coordination of the study and revised the manuscript in form and content. All authors read and approved the final manuscript.
